# A243 CILIATED PANCREATIC FOREGUT CYST MIMICKING A MUCINOUS CYSTIC LESION: A CASE REPORT

**DOI:** 10.1093/jcag/gwae059.243

**Published:** 2025-02-10

**Authors:** C Roda, J Telford, K Berg, D Sanders

**Affiliations:** Gastroenterology, The University of British Columbia Faculty of Medicine, Vancouver, BC, Canada; St Paul’s Hospital, Vancouver, BC, Canada; Kelowna General Hospital, Kelowna, BC, Canada; Kelowna General Hospital, Kelowna, BC, Canada

## Abstract

**Background:**

Ciliated foregut cysts are rare congenital cysts that arise from the embryonic foregut and tracheobronchial tree, and are composed of an inner ciliated epithelium. While most commonly found in the mediastinum, they can also occur in the hepatopancreaticobiliary system. To our knowledge there has been less than 10 case reports describing ciliated pancreatic foregut cysts.

**Aims:**

Report a case of a ciliated pancreatic foregut cyst and its associated imaging characteristics, cyst fluid analysis, and cytology.

**Methods:**

A retrospective case report.

**Results:**

A 66 year old female with invasive ductal adenocarcinoma of the right breast diagnosed in 2016 was found incidentally during staging work-up to have a 6.9 x 5.8 cm pancreatic tail cyst with peripheral calcifications on computed tomography scan of the abdomen. She had no history of pancreatitis or family history of pancreatic cancer, was a non-smoker, consumed minimal alcohol, and had normal liver enzymes.

An endoscopic ultrasound (EUS) with fine needle aspiration (FNA) was performed 02/2017 showing normal pancreatic parenchyma with a 7 cm anechoic simple pancreatic tail cyst without septations, mural nodularity or dilation of the pancreatic duct. Cyst fluid analysis showed an amylase of 9,174 U/L and carcinoembryonic antigen (CEA) of 1,731mg/L in keeping with a mucinous cystic lesion. The patient was subsequently followed by magnetic resonance imaging of the pancreas every 6 - 12 months for 5 years with ongoing cyst stability (5.2 x 4.9 x 5.7 cm on 06/2017 and 5.8 x 3.8 x 4.8 cm on 09/2021). Three subsequent EUS with FNA were performed between 02/2022 - 09/2023 showing constancy in cyst characteristics. Cyst fluid CEA remained >200 mg/L and cytologic evaluation on two occasions was non revealing (Table 1).

The working diagnosis was a side branch intraductal papillary mucinous neoplasm. Given concern for malignant potential, a laparoscopic distal pancreatectomy with splenectomy was performed 02/2024. Surgical pathology showed a unilocular cyst with serious fluid and an epithelial lining with a ciliated boarder positive for CK7 and negative for ER. Findings were diagnostic of a congenital ciliated foregut cyst.

**Conclusions:**

Our case report is the first to show EUS FNA cyst fluid analysis overtime for a ciliated pancreatic foregut cyst. The cysts fluid mimics that of a mucinous cystic lesion. Obtaining adequate cytology is paramount for identifying ciliated epithelium in order to differentiate these benign congenital cysts from those with malignant potential.

Table 1. Endoscopic ultrasound fine needle aspiration cyst fluid analysis from a ciliated pancreatic foregut cyst

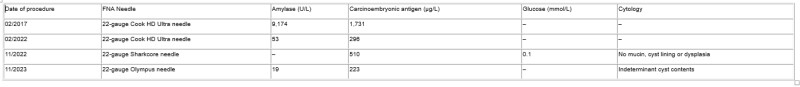

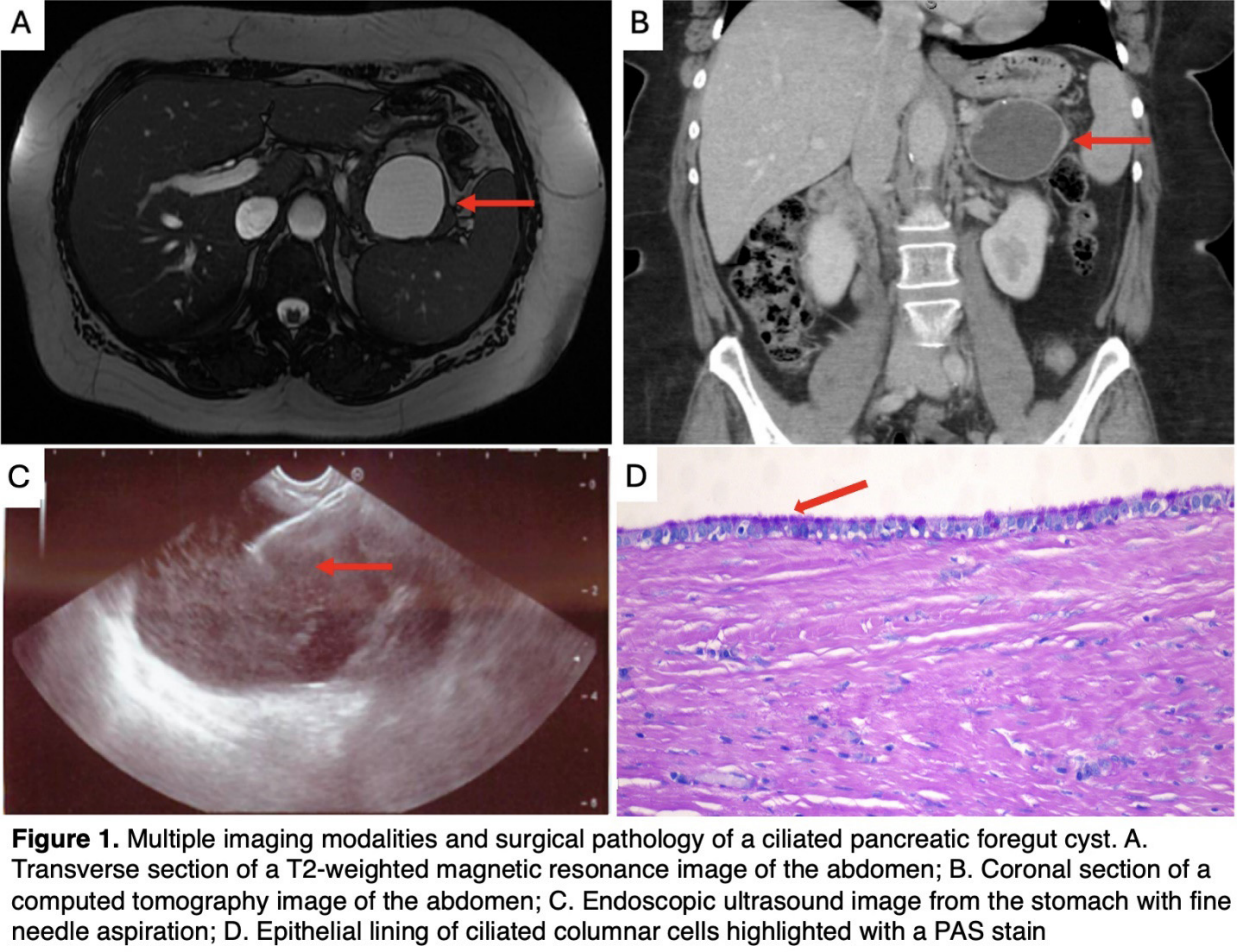

**Funding Agencies:**

None

